# Association of Domain-Specific Physical Activities with Non-Alcoholic Fatty Liver Disease in Workers: A Focus on Gender Differences

**DOI:** 10.3390/metabo16070454

**Published:** 2026-06-28

**Authors:** Seong-Uk Baek, Jin-Ha Yoon

**Affiliations:** 1 Graduate School, Yonsei University College of Medicine, Seoul 03722, Republic of Korea; 2 Department of Preventive Medicine, Yonsei University College of Medicine, Seoul 03722, Republic of Korea; 3 Institute for Innovation in Digital Healthcare, Yonsei University Health System, Seoul 03722, Republic of Korea; 4 The Institute for Occupational Health, Yonsei University College of Medicine, Seoul 03722, Republic of Korea

**Keywords:** exercise, health behaviors, hepatic steatosis, lifestyle, physical activity paradox, sex difference

## Abstract

**Objectives:** Occupational physical activity (OPA) and leisure-time physical activity (LTPA) have contrasting health effects, a phenomenon known as the “physical activity paradox.” We explored the domain-specific associations between physical activity and non-alcoholic fatty liver disease (NAFLD). **Methods:** This cross-sectional study included 20,584 Korean workers (10,846 women). Physical activity was assessed using the Global Physical Activity Questionnaire, and NAFLD was assessed using the hepatic steatosis index and the presence of metabolic dysfunction. Logistic regression models were employed to explore the association between each domain of physical activity and NAFLD. The associations are presented as odds ratios (ORs) and 95% confidence intervals (CIs). **Results:** The prevalence of NAFLD was 30.6% in men and 18.1% in women. For male workers, ≥300 min/week of OPA was positively associated with NAFLD (OR: 1.41; 95% CI: 1.15–1.72), while ≥300 min/week of LTPA was negatively associated with NAFLD (OR: 0.79; 95% CI: 0.67–0.93). In female workers, LTPA was negatively associated with NAFLD from a lower level (OR: 0.63; 95% CI: 0.52–0.78 for 1–149 min/week; OR: 0.71; 95% CI: 0.56–0.89 for 150–299 min/week; OR: 0.58; 95% CI: 0.43–0.78 for ≥300 min/week of LTPA), while OPA had no clear association with NAFLD. **Conclusions:** OPA and LTPA were differentially associated with NAFLD in workers. Domain- and sex-specific effects of physical activity should be considered for the prevention and management of NAFLD.

## 1. Introduction

Promotion of physical activity (PA) is vital in public health policies. Active engagement in PA can decrease the risk of mortality, cardiovascular disease, and metabolic dysfunction [1]. Consequently, the current PA guidelines recommend ≥150 min/week of moderate-to-vigorous PA (MVPA) for adults [2,3]. However, growing evidence suggests that the health impacts of PA vary depending on its domain. For example, recent studies have demonstrated that, although leisure-time PA (LTPA) reduces the risk of mortality or cardiovascular diseases, occupational PA (OPA) is not associated or even positively associated with these outcomes [4,5]. Additionally, previous studies have found that OPA, in comparison with LTPA, has no beneficial effects on metabolic dysfunction, such as diabetes mellitus [6], insulin resistance [7], and obesity [8]. These contrasting health effects of OPA and LTPA, also known as the “PA paradox,” have drawn significant academic interest in the field of occupational and public health in recent years.

Globally, along with the steady increase in metabolic syndrome and obesity, public health concerns related to non-alcoholic fatty liver disease (NAFLD) have been increasing [9], with approximately 15% of the Korean population estimated to be affected by it [10]. NAFLD is a well-documented manifestation of metabolic dysfunction; thus, PA is known to have protective effects on the prevention of NAFLD. Engagement in PA reduces the delivery of free fatty acids for fatty acid synthesis within the liver, leading to a reduction in liver fat content inflammation [11]. A meta-analysis of epidemiological studies has also confirmed that engaging in PA can reduce the risk of incident NAFLD [12]. Therefore, promoting PA is pivotal in prevention and treatment strategies for NAFLD.

Despite the evidence uncovered over the past several decades regarding the relationship between PA and NAFLD, there remain research gaps deserving of further investigation. First, little is known about whether OPA and LTPA have differential associations with NAFLD. A recent study based on the United States (US) population suggested that, while LTPA is associated with a reduced risk of NAFLD in a dose–response manner, OPA has no clear association with NAFLD [13]. In contrast, a previous Korean study found that 150–299 min/week of OPA and ≥300 min/week of LTPA are both associated with a reduced risk of NAFLD [14]. Second, the current body of literature concerning the association between OPA and LTPA does not sufficiently address the aspect of sex differences within this relation. Indeed, sex differences in the health effects of OPA and LTPA within the context of the “PA paradox” phenomenon have been underscored as an important yet underexplored research agenda [15,16]. A recent study showed that OPA reduces cardiovascular risk in female workers but acts as a risk factor for male workers [17]. Considering the differences in the hormonal profile, metabolism, and physical capabilities of the sexes, the physical effects of PA can manifest differently in men and women [18]. Therefore, this study aimed to explore how each domain of PA is differentially associated with NAFLD and whether there are potential differences in this relation between men and women, based on a nationally representative sample of Korean workers.

## 2. Methods

### 2.1. Study Sample

The study sample was obtained from the 2014 to 2021 Korea National Health and Nutrition Examination Survey (KNHANES), which is an annual, nationwide study with a repeated cross-sectional design [19]. The Korea Disease Control and Prevention Agency (KDCA) utilized a multistage cluster sampling method to select a nationally representative sample of the Korean population. In this approach, enumeration districts in Korea served as primary sampling units, and households within each district served as secondary sampling units. The survey involved face-to-face interviews conducted by professional interviewers employed by the KDCA. We included survey participants from the year 2014 because this was the year in which relevant information on OPA and LTPA was first collected.

**Figure 1:**
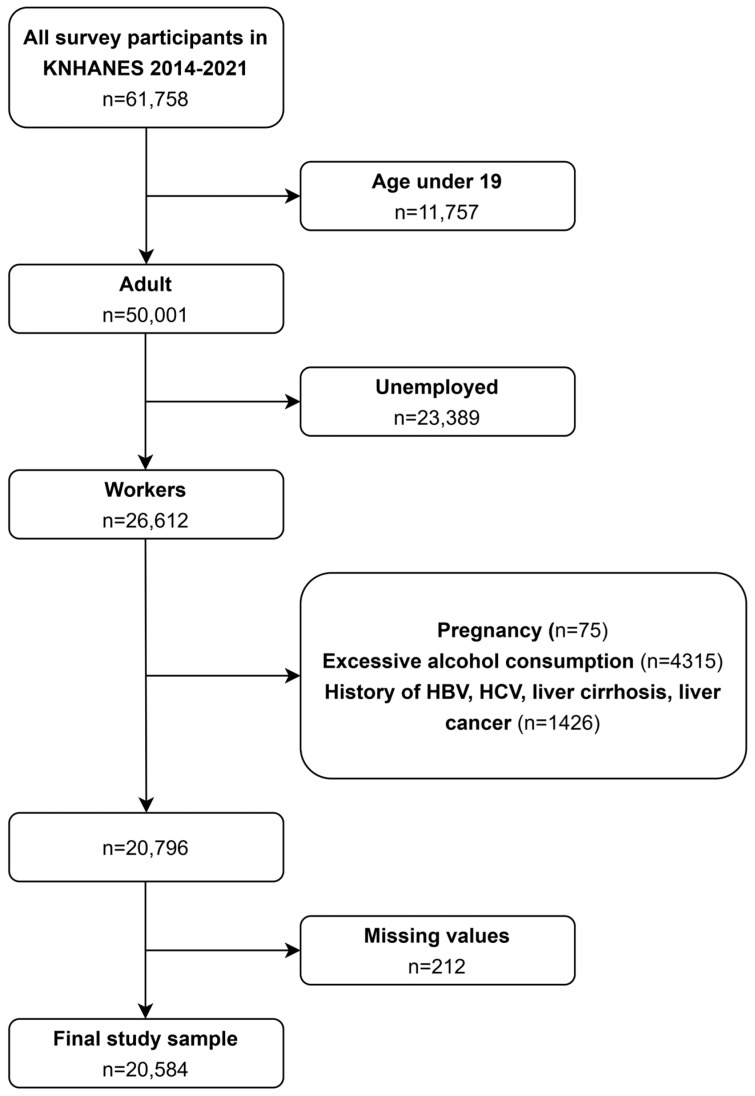
Flowchart of the selection process of the study sample (KNHANES: Korea National Health and Nutrition Examination Survey).

The study sample selection process is illustrated in Figure 1. In total, 61,758 participants were included in the initial study sample. Subsequently, we restricted the study sample using the following criteria: (i) adults (aged ≥19); (ii) individuals engaged in economic activities (workers); (iii) individuals with no significant alcohol consumption (>140 g/week for men and >70 g/week for women); (iv) those with no history of liver diseases, including hepatitis B or C, liver cirrhosis, and liver cancer; and (v) absence of missing values. After refinement of the sample, 20,584 adult workers (9738 men and 10,846 women) were included in the statistical analyses.

### 2.2. Data Availability and Ethics Statement

The KNHANES datasets were made publicly available following the anonymization process of the KDCA (https://knhanes.kdca.go.kr/knhanes, accessed on 12 January 2023). The 2014–2021 KNHANES was conducted with the ethical approval of the Institutional Review Board of the KCDA (2013-12EXP-03-5C; 2018-01-03-P-A; 2018-01-03-C-A; 2018-01-03-2C-A; 2018-01-03-5C-A).

### 2.3. Variables

#### 2.3.1. PA

Physical activity was assessed using the Korean version of the Global Physical Activity Questionnaire (GPAQ) [20], which was originally developed by the World Health Organization (WHO) and then adapted for use in Korea [21]. The Korean GPAQ (K-GPAQ) was demonstrated to be a reliable and valid measurement tool for measuring PA levels in the Korean population [21]. The following three main domains of PA, OPA, LTPA, and transportation-related PA (TRPA) were assessed, along with information on the intensity (moderate or vigorous), frequency (number of PA sessions per week), and duration (min per session). For OPA, the participants were asked, “Does your job include PA of moderate intensity, such as brisk walking, carrying light objects, cleaning, and caregiving, which lead to a slight increase in your breathing and heart rate for at least 10 min?” (moderate OPA) and “Do you regularly participate in sports, exercises, or leisure activities with high intensity, such as lifting and carrying heavy objects (≥20 kg), digging, labor at construction sites, and transporting items upstairs, which lead to panting and a significant elevation in heart rate for at least 10 min?” (vigorous OPA). For LTPA, the participants were asked, “Do you regularly engage in sports, exercises, or leisure activities of moderate intensity, such as brisk walking, gentle jogging, weightlifting, golf, or dance, which lead to a slight increase in your breathing and heart rate for at least 10 min?” (moderate LTPA) and “Do you regularly engage in sports, exercises, or leisure activities with high intensity, such as running, jumping rope, hiking, playing basketball, swimming, or badminton, which lead to panting and a significant elevation in heart rate for at least 10 min?” (vigorous LTPA). For TRPA, participants were asked, “When you move from one place to another for activities, such as work, shopping, grocery shopping, attending worship services, going to school, or commuting to academic institutes, etc., do you typically engage in walking or cycling for a continuous period of at least 10 min?” TRPA was regarded as moderate-intensity PA, according to the WHO GPAQ analysis guide [22]. Subsequently, the amount of MVPA per week was calculated as follows for each domain [21,22]:

$$MVPA\;\left(\min/week\right)=moderate\;PA\;\left(\min/week\right)+2\times vigorous\;PA\;\left(\min/week\right)$$

The amount of vigorous PA was doubled based on the WHO GAPQ analysis guidelines [22], which considers 1 min of vigorous PA to be metabolically equivalent to 2 min of moderate PA. The total PA (TPA) (MVPA min/week) was calculated as the sum of OPA, LTPA, and TRPA. Subsequently, workers’ PA levels were classified as inactive (0 min/week of MVPA), insufficiently active (1–150 min/week), sufficiently active (150–299 min/week), or highly active (≥300 min/week) for TPA, OPA, LTPA, and TRPA, in accordance with the recommendations outlined in the current PA guidelines [2,3].

#### 2.3.2. NAFLD

NAFLD was defined using the hepatic steatosis index (HSI), a validated methodology used for predicting fatty liver conditions [23]. Developed as a noninvasive screening tool for NAFLD in the Korean population, the HSI utilizes the ratio of serum alanine aminotransferase (ALT) to aspartate aminotransferase (AST), body mass index (BMI), presence of diabetes of mellitus, and sex in its calculation [23].

$$HSI=8\times \left(\frac{ALT}{AST}\right)+BMI\;(kg/m^{2})+2\;\left(if\;diabetes\;mellitus\right)+2\;(if\;female)$$

The HSI was developed based on the Korean population and has been shown to have a reasonable accuracy in classifying NAFLD in previous studies (area under the receiver operating characteristic curve: 0.82–0.86) [23,24]. In this study, individuals were defined as having diabetes mellitus if they met one or more of the following criteria: (i) fasting glucose ≥126 mg/dL; (ii) glycated hemoglobin ≥6.5% mg/dL; and (iii) current use of insulin or antidiabetic oral agents. Individuals with an HSI of ≥36 were considered to have NAFLD [23]. As supplementary measurements for classifying NAFLD, the K-NAFLD score [25] and the ZJU index [26] were utilized in the sensitivity analyses (Table S1).

#### 2.3.3. Confounders

The following confounders were adjusted in our regression models: sex (“male” and “female”), age (“19–29,” “30–39,” “40–49,” “50–59,” and “≥60” years), residential region (“urban” and “rural”), educational level (“middle school or below,” “high school,” and “college or above”), income level categorized based on quartile values of monthly income for each survey year (Q1–Q4), marital status (“married” and “unmarried/others”), occupation type categorized based on the Korean Standard Classification of Occupation (“white collar,” “service/sales worker,” and “blue collar”), current smoking status (“yes” and “no”), and survey year.

### 2.4. Statistical Analysis

For the descriptive analysis, we first explored the characteristics of the study participants according to the amount of TPA (MVPA min/week). Subsequently, the prevalence of NAFLD was calculated based on the amount of PA in each domain (TPA, OPA, LTPA, and TRPA).

In our regression analysis, we initially examined the relation between the amount of TPA and NAFLD (Model A), followed by an exploration of the associations of OPA, LTPA, and TRPA with NAFLD (Model B). Subsequently, we incorporated interaction terms between sex and each PA domain (sex × OPA + sex × LTPA + sex × TRPA) into Model B, allowing for variations in the association between each PA domain and NAFLD across sexes. The outcomes of this model were presented as the associations of OPA, LTPA, and TRPA with NAFLD within each sex stratum. For interaction *p*-values, in addition to traditional *p* values, false discovery rate (FDR)-corrected *p* values were reported to address multiple testing within each model. Consistent with previous conventions, *p*-values < 0.05 were interpreted as statistically significant, *p*-values between 0.05 and 0.10 as marginally significant, and *p*-values ≥ 0.10 as non-significant.

Sensitivity analyses were conducted to verify the robustness of the findings. We employed alternative measurements for NAFLD classification (the K-NAFLD score and ZJU index) to investigate whether a similar pattern could be observed. All statistical analyses and visualizations were performed using R software (version 4.2.3; R Foundation for Statistical Computing, Vienna, Austria). In all statistical analyses, the complex survey design of the KNHANES was considered using the R package “survey.” Logistic regressions were used to estimate odds ratios (ORs) and their corresponding 95% confidence intervals (CIs) using the “svyglm” function in the “survey” package.

## 3. Results

Table 1 shows the distribution of the study participants according to the amount of TPA. Among the study participants, 29.7%, 21.5%, 20.1%, and 28.8% showed inactive, insufficiently active, sufficiently active, and highly active TPA levels, respectively. Individuals with the highest TPA levels were more likely to be male, younger, possess higher educational and income levels, and be employed in nonblue-collar occupations than those with the lowest TPA levels.

In Table 2, which presents the results from the regression models, including sex interactions, there was a negative interaction between female sex and highly active OPA (*p* = 0.019), and between female sex and insufficiently active LTPA (*p* = 0.002, FDR-corrected *p* = 0.018). Among the male workers, the adjusted OR for the association between OPA and NAFLD was 1.41 (1.15–1.71) for the highly active group compared to the inactive OPA group. The adjusted OR for the association between LTPA and NAFLD was 0.79 (0.67–0.93) for the highly active group compared to the inactive LTPA group. Among the female workers, the adjusted OR for the association between LTPA and NAFLD was 0.63 (0.52–0.78) for the insufficiently active group, 0.71 (0.56–0.89) for the sufficiently active group, and 0.58 (0.43–0.78) for the highly active group compared to the inactive LTPA group. No clear association was observed between OPA and NAFLD in the female workers. Additionally, no clear association was observed between TRPA and NAFLD in either sex. When using continuous PA variables (Table S2; per 60 min/week of MVPA), the ORs (95% CIs) for NAFLD among men were 1.01 (1.00–1.02) for OPA, 0.96 (0.92–0.99) for LTPA, and 1.00 (0.99–1.02) for TRPA. The corresponding ORs (95% CIs) among women were 0.99 (0.98–1.01), 0.94 (0.90–0.97), and 1.01 (0.99–1.03), respectively. The *p*-values for the interaction between sex and each physical activity domain were 0.222 for OPA, 0.040 for LTPA, and 0.289 for TRPA.

**Figure 2:**
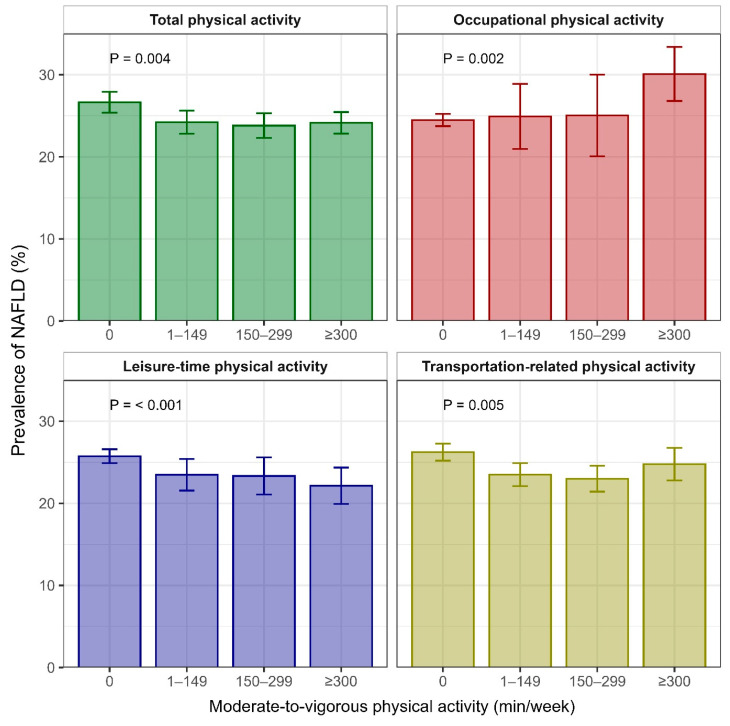
Prevalence of NAFLD according to total, occupational, leisure-time, and transportation-related physical activity. Cochran–Armitage trend tests were used to calculate *p* values. NAFLD: non-alcoholic fatty liver disease.

Figure 2 shows the prevalence of NAFLD according to each PA domain. The prevalence of NAFLD across different TPA levels was 26.7%, 24.2%, 23.8%, and 24.1% for the inactive, insufficiently active, sufficiently active, and highly active groups, respectively. Regarding OPA, the prevalence of NAFLD was 24.5%, 24.9%, 25.0%, and 30.1% in the inactive, insufficiently active, sufficiently active, and highly active groups, respectively. The prevalence of NAFLD for LTPA was 25.7%, 23.5%, 23.3%, and 22.1% in the inactive, insufficiently active, sufficiently active, and highly active groups, respectively. For TRPA, the prevalence of NAFLD was 26.2%, 23.5%, 23.0%, and 24.8% in the inactive, insufficiently active, sufficiently active, and highly active groups, respectively.

**Figure 3:**
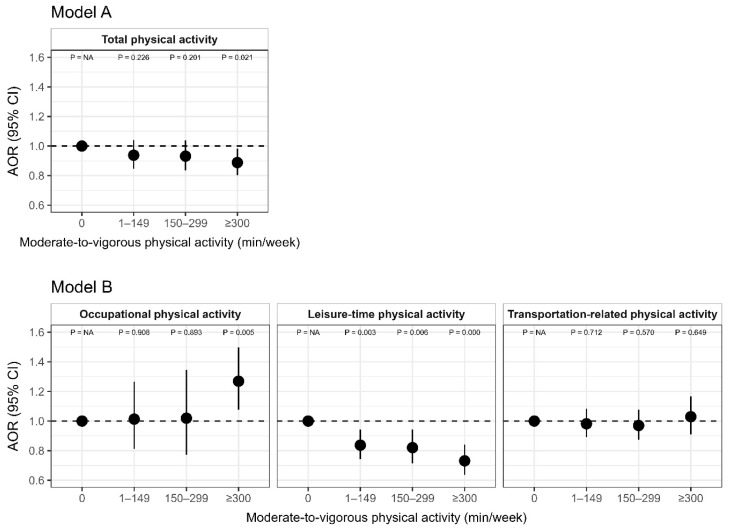
Association between total and domain-specific physical activities and NAFLD. Model A: Total physical activity was included as the main independent variable. Model B: Domain-specific physical activity variables were included as the main independent variables. AOR, adjusted odds ratio.

The association between each PA domain and NAFLD is shown in Figure 3. In Model A, the adjusted OR (95% CI) for the association between TPA and NAFLD was 0.94 (0.85–1.04) for the insufficiently active group, 0.93 (0.84–1.04) for the sufficiently active group, and 0.89 (0.80–0.98) for the highly active group compared to the inactive group. In Model B, where OPA, LTPA, and TRPA were included as independent variables, highly active OPA exhibited a positive association with NAFLD (OR: 1.27; 95% CI: 1.08–1.50) compared to the inactive OPA group, while LTPA showed a negative association with NAFLD (OR: 0.84; 95% CI: 0.74–0.94 for the insufficiently active group; OR: 0.82; 95% CI: 0.71–0.94 for the sufficiently active group; OR: 0.73; 95% CI: 0.64–0.84 for the highly active group) compared to the inactive LTPA group. TRPA was not associated with NAFLD.

The results of the sensitivity analyses, employing alternative measurements for the NAFLD classification, are comparable to those shown in Table 2, with a higher OR for NAFLD in males engaged in highly active OPA and a lower OR for NAFLD in females engaged in insufficiently to highly active LTPA levels (Table 3).

## 4. Discussion

In this study, we observed divergent associations between OPA, LTPA, and NAFLD in Korean workers. Among the male workers, engaging in highly active OPA was positively associated with NAFLD, whereas engaging in highly active LTPA was negatively associated with NAFLD. In the female workers, the beneficial effect of LTPA was observed from a lower level (≥1 min/week of MVPA), while OPA had no clear association with NAFLD. The absence of a significant association between OPA and NAFLD in women, as well as the lack of a clear association between TRPA and NAFLD, highlights the need for a more nuanced understanding of the relationship between PA and NAFLD that incorporates both sex- and domain-specific patterns beyond the existing PA paradox framework.

The results of our study are consistent with those of previous studies, which showed that engaging in LTPA is associated with a reduced risk of NAFLD. For example, recent studies conducted in the United Kingdom [27], US [13], and China [28] have shown that engagement in LTPA acts as a protective factor against NAFLD. Therefore, in current practice, the promotion of PA is of utmost importance for the prevention and management of NAFLD. It has been well-documented that weight reduction contributes to the remission of NAFLD, with overweight and obesity being the most significant risk factors for the development of NAFLD [29]. Moreover, studies have shown that the beneficial effects of PA, including a decrease in liver fat and various metabolic advantages, are independent of changes in body weight. An increase in insulin sensitivity and a decrease in circulatory lipids may underlie these effects [30,31].

However, it should be noted that the association between PA and NAFLD can vary according to its domain. Although the beneficial effects of LTPA on NAFLD have been well-documented in the existing literature, there is limited knowledge regarding the association between OPA and NAFLD. A previous study based on a US population showed that, although LTPA is associated with a reduced risk of NAFLD, OPA has no clear association with it [13]. In this study, we observed that ≥300 min/week of OPA was positively associated with NAFLD, especially in male workers. Our result can be seen as supporting the hypothesis of the “PA paradox,” which states that OPA and LTPA have contrasting effects on workers’ health. Although the exact mechanism remains unclear, significant differences exist between OPA and LTPA in terms of exercise intensity, context, and autonomy. Although individuals engage in LTPA with ample recovery time and diversity in position and movements, OPA often consists of repetitive and monotonous movements with a limited recovery time and reduced autonomy [32]. These characteristics can render OPA counterproductive for weight loss and metabolic improvement [33,34,35]; instead, OPA has been shown to induce systemic inflammation and an increase in blood pressure [36,37]. Therefore, our findings emphasize the need to distinguish between different domains of PA when assessing its health impacts and caution against considering OPA as a mere substitute for LTPA in the prevention and management of NAFLD.

Our study is one of the few to show how the associations of OPA and LTPA with NAFLD vary by sex. A previous study by Kim et al. showed that the association between LTPA and NAFLD was pronounced among women, while no significant sex interactions were observed for the associations of OPA and TRPA with NAFLD in a US population [38]. First, men and women exhibit a distinct physiological reaction to LTPA, owing to differences in lipid metabolism and muscle composition. Studies have suggested that the beneficial effect of LTPA on NAFLD can be stronger among women because women tend to have a greater quantity of type I muscle fibers, showing superior lipid oxidation capabilities and heightened sensitivity to insulin when compared to men’s muscle tissue [39,40]. Thus, even minimal levels of LTPA might exhibit a significant metabolic benefit for women in comparison to men. However, these proposed mechanisms remain speculative, and further research is needed to elucidate the roles of muscle fiber composition and inflammatory pathways in explaining the observed sex differences. Second, although OPA was assessed using the same survey questions for both sexes, the evaluation was based on subjective assessments of physiological responses to OPA, potentially leading to a situation in which female workers might have tended to overestimate the intensity of their OPA relative to male workers. Moreover, in the Korean labor market, there is substantial gender-based occupational segregation, with physically demanding roles such as working at construction sites or tasks requiring heavy lifting primarily being assigned to men [41]. As a result, men may have more physically demanding and strenuous OPA, even when their questionnaire responses categorize them in the same OPA group. This distinction could partially explain why the positive association between highly active OPA and NAFLD was observed only in male workers. For TRPA, an association with NAFLD was observed only in women with very high levels of activity (≥300 min/week of MVPA) and only when the ZJU index was used to define NAFLD. Although this finding was not consistently observed across models using other NAFLD indices and should therefore be interpreted with caution, it may be hypothesized that that excessive levels of TRPA do not necessarily confer additional metabolic benefits and may instead impose a greater physical burden among women.

Our study has several limitations. First, owing to the cross-sectional design of this study, we could not assert a temporal relation between each PA domain and NAFLD. There is a possibility of reverse causation, wherein individuals with better physical health are more inclined to participate in active OPA or LTPA than those with suboptimal health conditions. Individuals with preclinical or existing NAFLD may be more likely to reduce their LPTA and engage in occupations requiring less OPA. Therefore, reverse causation may have amplified the magnitude of the observed associations, potentially biasing the results away from the null. Second, the possibility of unmeasured confounding factors must be considered. For example, we were unable to consider several factors, including exposure to toxins, use of hepatotoxic medications, or genetic variants, owing to a lack of information. Additionally, detailed information on occupational exposures, such as shift work, ergonomic risk factors, and cumulative mechanical workload, was not considered in this study. These occupational factors may serve as important confounders in the association between occupational physical activity and NAFLD. Third, our study assessed NAFLD using noninvasive tools, which could have introduced misclassification errors. Although noninvasive methods have demonstrated reasonable accuracy in classifying NAFLD and have been widely employed in previous epidemiological studies, the results of our study would benefit from validation through future investigations using pathological or imaging modalities. Nonetheless, we conducted sensitivity analyses utilizing alternative noninvasive measurement tools, including the K-NAFLD score and ZJU index, and found that the main results remained consistent. Biomarker-based measurement tools, such as the HSI, exhibited only moderate accuracy for the classification of NAFLD [23,24], which may introduce non-differential misclassification and bias the estimated associations toward the null. Therefore, future studies should consider using imaging modalities, such as ultrasonography or transient elastography, or biopsy to improve the accuracy of the assessment. Fourth, our assessment of PA relied on self-reported data rather than on objective measurements. Although a previous study confirmed the validity of the K-GPAQ in capturing objectively measured PA levels [21], the potential for subjectivity in reporting the intensity and duration of PA and the influence of social desirability bias cannot be ruled out. Specifically, important characteristics of physical activity, such as intensity, posture, and recovery time, differ across PA domains but were not adequately captured by the GPAQ. For example, reporting bias related to PA may differ by sex, and the characteristics of occupational physical activity may also vary between men and women. Consequently, such nuanced patterns of PA are not fully captured by the GPAQ. Therefore, future studies should employ device-based measurements, such as accelerometers, to achieve a more accurate assessment of PA exposure. Sixth, although we discussed differential muscle fiber composition and reporting bias as potential explanations for the observed sex differences, these pathways could not be formally evaluated through mediation analyses incorporating inflammatory markers or adiposity. Therefore, these proposed mechanisms should be considered hypothetical. Future in-depth studies are needed to elucidate the biological and behavioral mechanisms underlying the associations between domain-specific PA and NAFLD, as well as the observed sex differences. Seventh, some sex- and domain-specific PA categories included relatively small numbers of participants, particularly women with high levels of OPA. Consequently, the statistical power to estimate the associations in these subgroups may have been limited, potentially contributing to unstable estimates.

## 5. Conclusions

In this study, we observed distinct associations of OPA, LTPA, and TRPA with NAFLD in Korean workers. Our analysis showed that LTPA was negatively associated with NAFLD, whereas OPA was positively associated with NAFLD. Additionally, the association of OPA and LTPA with NAFLD varied according to sex, with a negative relation between LTPA and NAFLD being more prominent among female workers, whereas a positive association between OPA and NAFLD was observed only in male workers. Considering that the observed associations cannot be interpreted as causal because of the potential for reverse causation and residual confounding from unmeasured factors, such as dietary intake, sleep, and occupational stress, future longitudinal studies incorporating a broader range of relevant covariates are warranted to clarify the temporal relationships. Further research is also needed to elucidate the biological mechanisms underlying the observed sex differences, including the potential roles of muscle fiber composition and inflammatory pathways. Additionally, given the limitations of biomarker-based classification of NAFLD, further studies incorporating imaging modalities or liver biopsy are warranted to improve the accuracy of NAFLD assessment.

## Figures and Tables

**Table 1:** Baseline characteristics of study participants according to the amount of MVPA per week.

	Total Physical Activity (MVPA min/Week)			
	Inactive (0)	Insufficiently Active (1–149)	Sufficiently Active (150–299)	Highly Active (≥300)
**Sex**				
Male	2995 (52.1)	1938 (48.9)	1747 (49.2)	3058 (62.6)
Female	3563 (47.9)	2584 (51.1)	2345 (50.8)	2354 (37.4)
**Age**				
19–29	398 (9.1)	514 (15.0)	589 (19.4)	961 (23.7)
30–39	1004 (18.5)	745 (19.5)	715 (19.8)	1065 (21.8)
40–49	1435 (24.6)	1006 (23.7)	938 (24.2)	1222 (23.3)
50–59	1631 (25.9)	1068 (24.0)	879 (20.8)	1094 (19.1)
≥60	2090 (21.9)	1189 (17.8)	971 (15.8)	1070 (12.1)
**Region**				
Urban	4688 (77.6)	3691 (85.0)	3510 (88.8)	4559 (86.7)
Rural	1870 (22.4)	831 (15.0)	582 (11.2)	853 (13.3)
**Education level**				
Middle school or below	2052 (23.5)	1112 (18.4)	890 (15.8)	847 (10.8)
High school	2077 (34.2)	1417 (32.7)	1290 (33.4)	1924 (37.2)
College or above	2429 (42.3)	1993 (48.8)	1912 (50.8)	2641 (52.0)
**Income level**				
Q1	1381 (21.5)	945 (21.3)	783 (19.5)	1028 (19.4)
Q2	1748 (26.4)	1131 (24.8)	1116 (27.2)	1321 (24.6)
Q3	1786 (27.1)	1234 (27.1)	1056 (26.1)	1471 (27.3)
Q4	1643 (25.0)	1212 (26.9)	1137 (27.3)	1592 (28.7)
**Marital status**				
Married	5015 (74.8)	3229 (69.6)	2767 (64.2)	3637 (62.4)
Unmarried/others	1543 (25.2)	1293 (30.4)	1325 (35.8)	1775 (37.6)
**Occupation**				
White collar	2304 (38.8)	1915 (45.9)	1852 (48.8)	2387 (46.5)
Services/sales worker	1364 (21.1)	915 (20.1)	908 (22.4)	1147 (21.1)
Blue collar	2890 (40.0)	1692 (34.1)	1332 (28.8)	1878 (32.4)
**Smoking**				
No	5487 (80.7)	3951 (85.4)	3606 (86.1)	4567 (82.1)
Yes	1071 (19.3)	571 (14.6)	486 (13.9)	845 (17.9)

MVPA: moderate-to-vigorous physical activity. Values are presented as *n* (weighted %).

**Table 2:** ORs of NAFLD according to the level of physical activity in each domain and sex in a model with an interaction term between physical activity and sex.

	Amount of Physical Activity (MVPA min/Week)			
	Inactive (0)	Insufficiently Active (1–149)	Sufficiently Active (150–299)	Highly Active (≥300)
	OR (95% CI)	OR (95% CI)	OR (95% CI)	OR (95% CI)
**Male**				
OPA (OR)	Reference	1.04 (0.79–1.37)	0.99 (0.70–1.40)	1.41 (1.15–1.72)
OPA (*p* value)		0.756	0.940	0.001
LTPA (OR)	Reference	0.95 (0.82–1.11)	0.95 (0.82–1.11)	0.79 (0.67–0.93)
LTPA (*p* value)		0.530	0.161	0.005
TRPA (OR)	Reference	1.06 (0.92–1.21)	0.95 (0.82–1.10)	0.99 (0.84–1.17)
TRPA (*p* value)		0.421	0.492	0.944
**Female**				
OPA (OR)	Reference	0.97 (0.68–1.38)	1.14 (0.75–1.74)	0.87 (0.63–1.22)
OPA (*p* value)		0.865	0.531	0.425
LTPA (OR)	Reference	0.63 (0.52–0.78)	0.71 (0.56–0.89)	0.58 (0.43–0.78)
LTPA (*p* value)		<0.001	0.004	<0.001
TRPA (OR)	Reference	0.88 (0.76–1.01)	0.98 (0.85–1.14)	1.10 (0.91–1.32)
TRPA (*p* value)		0.068	0.818	0.323
**Interaction terms (*p* value)**				
Sex × OPA (FDR-corrected *p*)		0.739 (0.758)	0.600 (0.758)	0.019 (0.086)
Sex × LTPA (FDR-corrected *p*)		0.002 (0.018)	0.126 (0.227)	0.074 (0.167)
Sex × TRPA (FDR-corrected *p*)		0.061 (0.167)	0.758 (0.758)	0.440 (0.660)

NAFLD: non-alcoholic fatty liver disease; MVPA: moderate-to-vigorous physical activity; OPA: occupational physical activity; LTPA: leisure-time physical activity; TRPA: transportation-related physical activity; AOR: adjusted odds ratio; CI: confidence interval. The models were adjusted for sex, age, region, education, income, marital status, occupation, smoking status, and survey year.

**Table 3:** ORs of NAFLD according to level of physical activity in each domain and sex in a model with an interaction term between physical activity and sex using the K-NAFLD or ZJU index.

	K-NAFLD Index	ZJU Index
	OR (95% CI)	OR (95% CI)
**Male**		
**OPA (MVPA min/week)**		
Inactive (0)	1.00 (1.00–1.00)	1.00 (1.00–1.00)
Insufficiently active (1–149)	1.20 (0.88–1.63)	1.14 (0.86–1.50)
Sufficiently active (150–299)	0.78 (0.51–1.21)	1.03 (0.72–1.48)
Highly active (≥300)	1.28 (1.00–1.62)	1.34 (1.08–1.67)
**LTPA (MVPA min/week)**		
Inactive (0)	1.00 (1.00–1.00)	1.00 (1.00–1.00)
Insufficiently active (1–149)	0.96 (0.81–1.14)	1.05 (0.90–1.22)
Sufficiently active (150–299)	0.81 (0.66–0.99)	0.97 (0.81–1.16)
Highly active (≥300)	0.76 (0.62–0.93)	0.90 (0.76–1.07)
**TRPA (MVPA min/week)**		
Inactive (0)	1.00 (1.00–1.00)	1.00 (1.00–1.00)
Insufficiently active (1–149)	1.04 (0.89–1.21)	1.11 (0.96–1.27)
Sufficiently active (150–299)	0.87 (0.73–1.03)	0.90 (0.78–1.05)
Highly active (≥300)	0.95 (0.79–1.15)	1.02 (0.86–1.22)
**Female**		
**OPA (MVPA min/week)**		
Inactive (0)	1.00 (1.00–1.00)	1.00 (1.00–1.00)
Insufficiently active (1–149)	0.99 (0.62–1.58)	1.10 (0.80–1.52)
Sufficiently active (150–299)	1.56 (0.88–2.74)	1.07 (0.69–1.64)
Highly active (≥300)	0.75 (0.45–1.23)	0.72 (0.51–1.00)
**LTPA (MVPA min/week)**		
Inactive (0)	1.00 (1.00–1.00)	1.00 (1.00–1.00)
Insufficiently active (1–149)	0.60 (0.43–0.84)	0.63 (0.51–0.77)
Sufficiently active (150–299)	0.64 (0.45–0.91)	0.67 (0.53–0.85)
Highly active (≥300)	0.56 (0.36–0.89)	0.53 (0.39–0.71)
**TRPA (MVPA min/week)**		
Inactive (0)	1.00 (1.00–1.00)	1.00 (1.00–1.00)
Insufficiently active (1–149)	0.93 (0.76–1.13)	0.95 (0.83–1.10)
Sufficiently active (150–299)	0.95 (0.77–1.18)	1.06 (0.92–1.24)
Highly active (≥300)	1.21 (0.93–1.57)	1.29 (1.08–1.54)
**Interaction terms**		
Sex × insufficiently active OPA (FDR-corrected *p*)	0.493 (0.493)	0.890 (0.914)
Sex × sufficiently active OPA (FDR-corrected *p*)	0.060 (0.162)	0.914 (0.914)
Sex × highly active OPA (FDR-corrected *p*)	0.053 (0.162)	0.002 (0.006)
Sex × insufficiently active LTPA (FDR-corrected *p*)	0.014 (0.126)	<0.001 (0.006)
Sex × sufficiently active LTPA (FDR-corrected *p*)	0.249 (0.444)	0.015 (0.034)
Sex × highly active LTPA (FDR-corrected *p*)	0.217 (0.444)	0.002 (0.006)
Sex × insufficiently active TRPA (FDR-corrected *p*)	0.364 (0.493)	0.130 (0.167)
Sex × sufficiently active TRPA (FDR-corrected *p*)	0.393 (0.493)	0.122 (0.167)
Sex × highly active TRPA (FDR-corrected *p*)	0.108 (0.243)	0.056 (0.101)

NAFLD: non-alcoholic fatty liver disease; MVPA: moderate-to-vigorous physical activity; OPA: occupational physical activity; LTPA: leisure-time physical activity; TRPA: transportation-related physical activity; AOR: adjusted odds ratio; CI: confidence interval; FDR: false discovery rate. The models were adjusted for sex, age, region, education, income, marital status, occupation, smoking status, and survey year.

## Data Availability

The raw data can be obtained at https://knhanes.kdca.go.kr (accessed on 12 January 2023).

## Supplementary Materials

The following supporting information can be downloaded at: https://www.mdpi.com/article/10.3390/metabo16070454/s1, Table S1: Alternative criteria for the classification of NAFLD; Table S2: ORs of NAFLD according to level of each domain of physical activity and sex in a model with an interaction term between physical activity and sex. A model using continuous PA variables.
