# Supplementary material for: Association of Domain-Specific Physical Activities with Non-Alcoholic Fatty Liver Disease in Workers: A Focus on Gender Differences
Source: Metabolites. 2026 Jun 28;16(7):454. doi: 10.3390/metabo16070454 (PMC13413609; doi:10.3390/metabo16070454)
Supplement: Supplementary file 1 [file metabolites-16-00454-s001.zip › metabolites-4315302-supplementary.pdf]

Table S1. Alternative criteria for the classification of NAFLD.

Measures	Equation	Cutoff
K-NAFLD score[1]	0.913 × sex (2, if female; 1, if male) + 0.089 × WC + 0.032 × (systolic blood pressure + fasting glucose [mg/dL]) + TG (mg/dL) × 0.007 + ALT (IU/L) × 0.105 − 20.929	> 0.884
ZJU index[2]	BMI (kg/m^2) + fasting glucose (mmol/L) + TG (mmol/L) + 3 × (ALT/AST) + 2 (if female)	> 38

[1] Jeong S, Kim K, Chang J, Choi S, Kim SM, Son JS, Lee G, Kim W, Park SM. Development of a simple nonalcoholic fatty liver disease scoring system indicative of metabolic risks and insulin resistance. Ann Transl Med. 2020 Nov;8(21):1414

[2] Wang J, Xu C, Xun Y, Lu Z, Shi J, Yu C, Li Y. ZJU index: a novel model for predicting nonalcoholic fatty liver disease in a Chinese population. Sci Rep. 2015 Nov 16;5:16494.

Table S2. ORs of NAFLD according to level of each domain of physical activity and sex in a model with an interaction term between physical activity and sex. A model using continuous PA variables.

	**OR (95% CI)**
**Male**	
**OPA (MVPA min/week)**	
Continuous scale (60 min)	1.01 (1.00–1.02)
**LTPA (MVPA min/week)**	
Continuous scale (60 min)	0.96 (0.92–0.99)
**TRPA (MVPA min/week)**	
Continuous scale (60 min)	1.00 (0.99–1.02)
**Female**	
**OPA (MVPA min/week)**	
Continuous scale (60 min)	0.99 (0.98–1.01)
**LTPA (MVPA min/week)**	
Continuous scale (60 min)	0.94 (0.90–0.97)
**TRPA (MVPA min/week)**	
Continuous scale (60 min)	1.01 (0.99–1.03)
**Interaction terms**	
Sex × OPA	0.222
Sex × LTPA	0.040
Sex × TRPA	0.289

NAFLD: non-alcoholic fatty liver disease; MVPA: moderate-to-vigorous physical activity; OPA: occupational physical activity; LTPA: leisure-time physical activity; TRPA: transportation-related physical activity; AOR: adjusted odds ratio; CI: confidence interval. The model adjusted for sex, age, region, education, income, marital status, occupation, smoking status, and survey year.
